# The Effect of Direct Quenching on the Microstructure and Mechanical Properties of NiCrMo and Cu-Bearing High-Strength Steels

**DOI:** 10.3390/ma17061397

**Published:** 2024-03-19

**Authors:** Naipeng Zhou, Feng Chai, Xiaobing Luo, Weiyi Wang, Feng Gao

**Affiliations:** Department of Structural Steels, Central Iron and Steel Research Institute, Beijing 100081, China

**Keywords:** direct quenching, strengthening mechanism, NiCrMo steel, Cu-bearing steel

## Abstract

In this work, two types of 590 MPa grade steels, composed of NiCrMo steel and Cu-bearing steel, were processed using traditional offline quenching and tempering and direct quenching (DQ) and tempering. The influence of DQ on microstructural evolution and strengthening mechanisms of these two types of steel was investigated. Grain refinement and dislocation density increase were determined by controlled rolling and following the DQ process in both two types of steel. In Cu-bearing steels, the refined grains and high-density dislocation further promoted the precipitation behavior of Cu-rich particles and alloyed carbides during the tempering treatment. Compared with traditionally quenched and tempered steels, NiCrMo steels after the direct quenching and tempering (DQT) process achieved 106 MPa higher yield strength through grain refinement strengthening and dislocation strengthening, while the Cu-bearing steels after the DQT process achieved 159 MPa higher yield strength through grain refinement strengthening, dislocation strengthening, and precipitation strengthening. The contribution degree of different strengthening mechanisms was quantitatively analyzed. Grain refinement also compensated for the toughness loss caused by the increase in dislocation, leading to an impact energy of 237 J and 248 J at −84 °C for NiCrMo and Cu-bearing steels after DQT, respectively.

## 1. Introduction

NiCrMo high-strength steels have been widely used in the field of shipbuilding and engineering fields such as mining and dredging due to their mechanical properties of high strength with good toughness and ductility [1]. In recent years, extensive welding during shipbuilding has increased the demand for hull steel’s weldability; hence, Cu-bearing high-strength low-alloy (HSLA) steel has been developed to meet the demand [2,3,4,5]. The precipitation of Cu-rich particles makes up for the loss in strength, which is caused by the decrease in the alloy element content. Nowadays, both 590 MPa grade NiCrMo steels and Cu-bearing steels have been applied in hull structures, leading to a significant reduction in the ship weight.

Conventional heat treatments of high-strength NiCrMo steels and Cu-bearing steels include austenitization, water quenching, and tempering, leading to microstructures consisting of tempered martensite/bainite and precipitates, which contribute to a significant increase in strength due to the precipitation hardening mechanism [6,7,8,9,10,11]. The austenitizing process is quite inefficient in terms of energy and resources. However, the newly developed direct quenching (DQ) process offers an effective way to produce high-strength steels through controlled rolling, online quenching, and tempering treatment. The DQ process reduces the cost by avoiding the reheating step and allows for reducing costly alloying elements by achieving mechanical properties with leaner chemistry [12,13,14]. Wang [15] introduced the application of the DQ process in TRIP steel, a type of advanced high-strength steel (AHSS) in the automobile industry, and superior tensile strength (880 MPa) and total elongation (28%) were obtained. Wang [16] carried out direct quenching and partitioning to improve the strength and ductility of 30Si2MnCrMoVE ultra-high-strength steel (UHSS). Ari [17] revealed the role of C, V, Ti, and B on the microstructure and mechanical properties of direct-quenched and tempered martensitic steel. Xie [18] investigated the synergistic effects of Nb and B in the forms of grain refinement strengthening by nano-sized TiN-Nb(C, N) or TiN-NbC during the rolling and direct quenching process and precipitation strengthening by NbC carbides during tempering. Sumit [19] studied the effect of varying silicon contents (1.5, 0.75, and 0.25 wt.%) and direct quenching and partitioning parameters on carbide formation and retained the austenite stabilization of medium-carbon steel. Dhua [20] reported that the DQ process enables saving costs and promotes weldability by achieving mechanical properties with lean chemistry.

As discussed above, DQ has not been applied in high-strength steels in the shipbuilding industry, and the discussion is mainly centered on the chemical composition. In this study, two types of 590 MPa grade steel, composed of NiCrMo steel and Cu-bearing steel, were processed using traditional offline quenching and tempering and direct quenching and tempering.

## 2. Experimental Procedures

### 2.1. Experimental Steel

Two types of 590 MPa grade steel, composed of NiCrMo steel and Cu-bearing steel, were used in the present study. The chemical compositions of NiCrMo and Cu-bearing steels are shown in Table 1. The non-recrystallization temperatures (*T_nr_*) of NiCrMo and Cu-bearing steels were determined as 797 °C and 889 °C based on the empirical equation proposed by Boratto, as shown in Equation (1):

(1) $$T_{nr}=877+464w\left(C\right)+6445w\left(Nb\right)-644\sqrt{w\left(Nb\right)}+732w\left(V\right)-230\sqrt{w\left(V\right)}+890w\left(Ti\right)+363w\left(Al\right)-357w(Si)$$

where *T_nr_* is the non-recrystallization temperature, °C; *w*(C), *w*(Nb), *w*(V), *w*(Ti), *w*(Al), and *w*(Si) are the mass fractions of C, Nb, V, Ti, Al, and Si, respectively, %.

Ingots were prepared in a vacuum induction furnace. Steels were forged into blanks with a cross-section of 120 mm × 150 mm, and the blanks were homogenized at 1150 °C for 2 h prior to the hot-rolling procedure. A two-stage thermomechanical rolling process was carried out with a rough rolling temperature of 1150–1000 °C, a total reduction ratio of 67.5%, finish rolling temperatures of 780 °C (NiCrMo steel) and 850 °C (Cu-bearing steel), and a final thickness of 12 mm. The hot-rolled plates were then quenched and tempered using two methods: Some plates were directly quenched with a cooling rate >50 °C/s and tempered at 660 °C for 2 h; some plates were air-cooled, reaustenitized at 900 °C for 1 h, and then water-quenched and tempered at 660 °C for 2 h. For convenience, the NiCrMo steel and Cu-bearing steel after direct quenching and reaustenitized quenching are designated as NiCrMo-DQ, NiCrMo-RQ, Cu-DQ, and Cu-RQ, respectively. The quenched steels after the subsequent tempering treatment are designated as NiCrMo-DQT, NiCrMo-RQT, Cu-DQT, and Cu-RQT, respectively.

### 2.2. Characterization

Round tensile specimens with a gauge length of 25 mm and a diameter of 5 mm were prepared for room temperature tensile testing, which was performed at a strain rate of 1.7 × 10^−2^ s^−1^; two individual samples were tested, and the average value was determined. The dimension of V-notch samples for Charpy impact testing was 10 mm × 10 mm × 55 mm. The samples were cooled to −84 °C and tested using an Instron Dynatup 9250 (INSTRON, Boston, MA, USA) Charpy impact tester. Three individual samples were tested, and the average value was determined.

The microstructure was examined using scanning electron microscopy (SEM, FEI Quanta 650, FEI Company, Hillsboro, OR, USA), electron back-scattered diffractometry (EBSD, JSM 7200F, JEOL, Tokyo, Japan), and transmission electron microscopy (TEM, H800, HITACHI, Tokyo, Japan). Specimens for SEM and EPMA were mechanically polished and then etched with a 4% Nital solution. Specimens for EBSD were electrochemically polished using a mixture of 10% perchloric acid and 90% ethanol. Thin foils for the TEM experiment were prepared by twin-jet polishing in a mixture of 5% perchloric acid and 95% ethanol at −20 °C. The dislocation density was determined by X-ray diffraction (XRD, Rigaku, D/Max-RB, Rigaku, Akishima-shi, Japan), and dislocation densities were calculated from the full width at half maximum (FWHM) of the diffraction peaks, combined with the modified Williamson–Hall method, as shown in Equation (2) [21]:

(2) $$\frac{{\left(\frac{2\delta cos\theta }{\lambda }-\frac{0.9}{D}\right)}^{2}}{{\left(\frac{2\delta sin\theta }{\lambda }\right)}^{2}}=M^{2}b^{2}\frac{\pi \rho }{2}\times 0.285\left[1-q\frac{h^{2}k^{2}+k^{2}l^{2}+l^{2}h^{2}}{{\left(h^{2}+k^{2}+l^{2}\right)}^{2}}\right]$$

(3) $$D=\frac{0.89\lambda }{\delta cos\theta }$$

where *δ* is the FHWM of the diffraction peak, *θ* is the diffraction angle, *λ* is the X-ray wavelength, *ρ* is the dislocation density, and *b* is the mode of the Burgers vector. In this experiment, the Cu target with a wavelength of 0.15405 nm was used for XRD detection. *D* is the average grain size, calculated according to the Scheele Equation (3); *M* is a constant depending on the effective outer cut-off radius of the dislocation, and in this study, *M* = 2; *h*, *k*, and *l* are the Miller indices for each diffraction peak; and *q* is the dislocation characteristic parameter.

## 3. Results

### 3.1. Mechanical Properties

**Figure 1:**
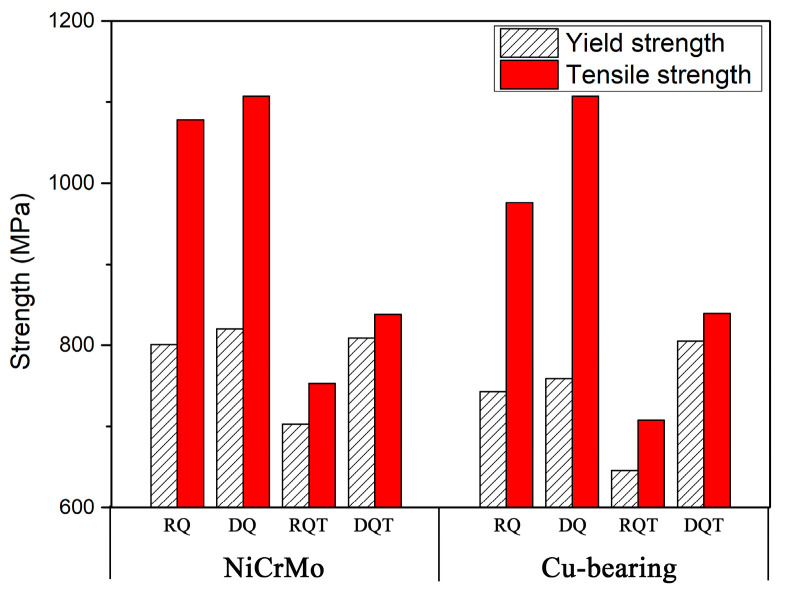
Strength comparison between NiCrMo and Cu-bearing steels.

Table 2 shows the experimental steels’ tensile properties and impact energy. The strength comparison of different states of NiCrMo and Cu-bearing steels is shown in Figure 1. As can be seen, the yield strength of all plates far exceeded the minimum stipulated requirement of 590 MPa. For the quenched steels, both the yield strength and tensile strength of DQ steels were slightly higher than those of RQ steels, while all exhibited a relatively low elongation. NiCrMo-DQ exhibited similar toughness to NiCrMo-RQ, while Cu-DQ exhibited a lower impact energy than Cu-RQ. The tempering process significantly improved the elongation and impact energy, leading to enhanced mechanical properties in tempered NiCrMo and Cu-bearing steels, with yield strength in the range of 646–809 MPa, tensile strength in the range of 708–839 MPa, elongation in the range of 20–25%, and impact energy in the range of 237–262 J at −84 °C.

In terms of the comparison between RQT and DQT plates, NiCrMo-RQT and NiCrMo-DQT exhibited similar low-temperature toughness, while Cu-RQT exhibited higher toughness than Cu-DQT. For both NiCrMo and Cu-bearing steels, the yield strength and tensile strength of DQT plates were still higher than those of RQT plates, indicating the significant strengthening effect of direct quenching. Remarkably, there was a higher degree of strengthening effect in Cu-bearing steels (159 MPa) than in NiCrMo steels (106 MPa).

### 3.2. Microstructures

**Figure 2:**
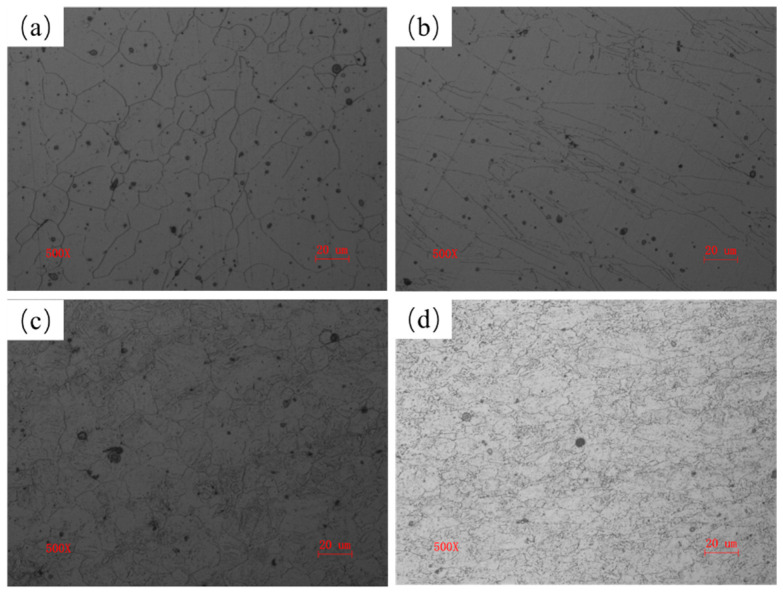
Prior austenite grains of (**a**) NiCrMo-RQ, (**b**) NiCrMo-DQ, (**c**) Cu-RQ, and (**d**) Cu-DQ.

The prior austenite grains of quenched steels are shown in Figure 2. Equiaxed grains were observed in RQ steels due to reaustenitization. DQ steels exhibited deformed and elongated grains due to the finish rolling stage under the non-recrystallization temperature and the subsequent direct quenching. The grain size of the prior austenite of NiCrMo-RQ, NiCrMo-DQ, Cu-RQ, and Cu-DQ was measured to be 14.4 μm, 8.8 μm, 10.1 μm, and 8.6 μm, respectively, indicating that the grain size refinement resulted from rolling in the recrystallization zone and fast cooling. There was a higher degree of grain refinement in NiCrMo steels resulting from the DQ process than in Cu-bearing steels.

**Figure 3:**
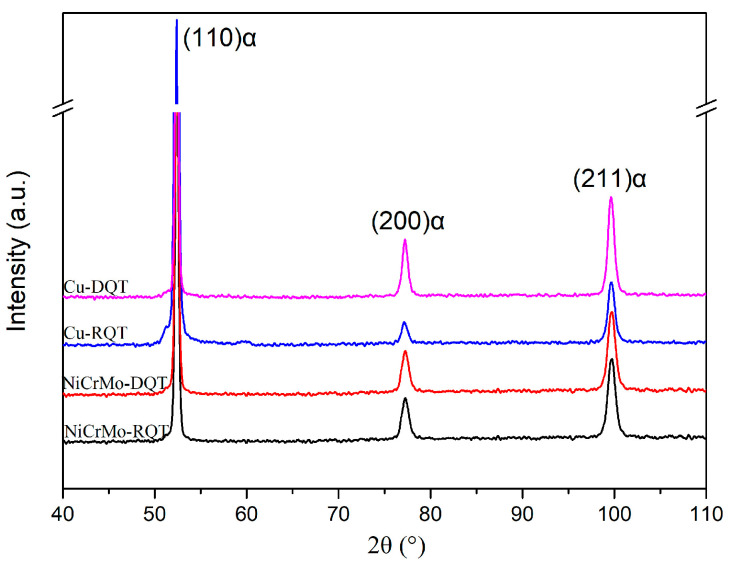
XRD results of quenched steels.

Besides the grain size, direct quenching may affect the dislocation density of steels. In DQ steels, a great number of dislocations were generated during the repeated deformation under the recrystallization zone and remained after subsequent fast cooling. The XRD test was conducted to quantitatively characterize the dislocation density of RQT and DQT steels, and the diffraction patterns are shown in Figure 3. Only the BCC phase was observed in the experimental steels, and the calculated dislocation densities of NiCrMo-RQT, NiCrMo-DQT, Cu-RQT, and Cu-DQT were 3.68 × 10^15^ m^−2^, 4.84 × 10^15^ m^−2^, 1.68 × 10^15^ m^−2^, and 2.21 × 10^15^ m^−2^, respectively.

**Figure 4:**
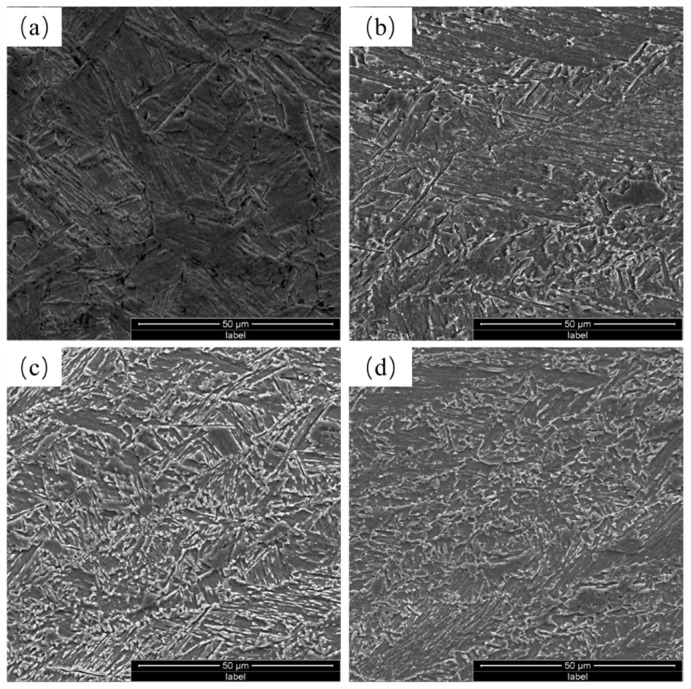
SEM images of the tempered steels: (**a**) NiCrMo-RQT, (**b**) NiCrMo-DQT, (**c**) Cu-RQT, and (**d**) Cu-DQT.

Figure 4 displays the representative SEM micrographs of the tempered steels. The equiaxed grains of RQT steels were inherited from RQ steels, while the elongated grains of DQT steels were inherited from the DQ steels. Lath martensite makes up the majority of the NiCrMo steels, and the Cu-bearing steels mainly consist of bainite. The presence of tiny carbides in NiCrMo steels suggests that the matrix was tempered martensite and a modest number of carbide precipitation can also be observed in aged Cu-bearing steels. The microstructure variation between the two types of steels is mainly attributed to different contents of hardenability elements such as Ni, Cr, and Mo.

**Figure 5:**
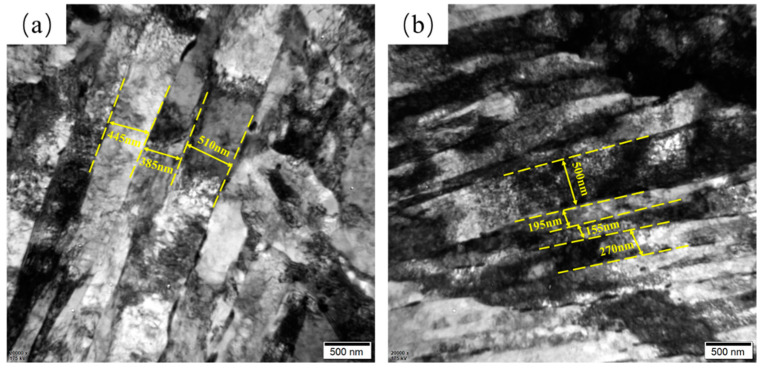
TEM images of (**a**) NiCrMo-RQT and (**b**) NiCrMo-DQT.

Figure 5 shows the martensitic lath morphology of the tempered NiCrMo steels. The average width of martensite lath was calculated from random positions of RQT and DQT steels in five TEM bright-field images. The average widths of NiCrMo-RQT and NiCrMo-DQT were 450 nm and 305 nm, respectively, indicating the grain refinement effect of the DQ process. The narrow martensite laths of NiCrMo-DQT resulted from controlled rolling and the subsequent direct quenching, consistent with the prior observation of austenite grains in the quenched steels. Moreover, higher dislocation density was found in the martensite lath in NiCrMo-DQT, which is consistent with the XRD calculation results.

### 3.3. Precipitation Behavior during Tempering

The precipitation behavior of Cu-rich particles and carbides can hardly be analyzed through SEM, so physicochemical phase analysis was carried out to quantitatively identify the carbides in the tempered steels, and the mass fraction is shown in Table 3. Two types of carbides, M3C and MC, were found in all tempered steels. The mass fraction of MC in NiCrMo-RQT, NiCrMo-DQT, Cu-RQT, and Cu-DQT was quite low and nearly the same, i.e., 0.063%, 0.066%, 0.061%, and 0.064%, respectively. The different constituents of MC between NiCrMo steels and Cu-bearing steels are in line with the two types of steels’ chemical composition. The mass fraction of M3C, which is the abbreviation of (Fe, Mn, Cr, Mo, Ni)3C, was relatively high. There were more M3C carbides in NiCrMo steels (0.838, 0.836) than in Cu-bearing steels (0.413, 0.454). By comparing the M3C content in NiCrMo and Cu-bearing steels, it can be concluded that the DQ process promotes the M3C precipitation only in the Cu-bearing steel.

**Figure 6:**
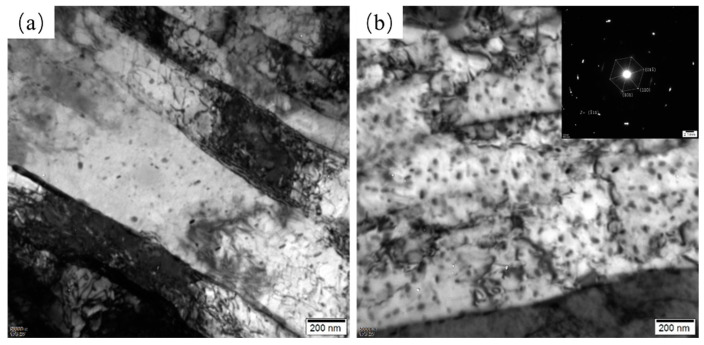
TEM images of precipitates in (**a**) Cu-RQT and (**b**) Cu-DQT.

**Figure 7:**
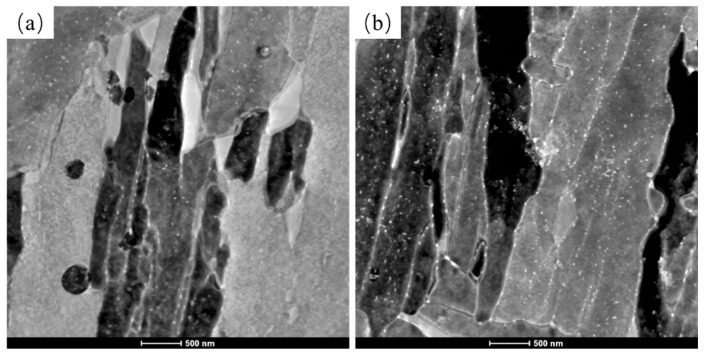
STEM images of (**a**) Cu-RQT and (**b**) Cu-DQT.

In Cu-bearing HSLA steels, the precipitation strengthening of nanoscale Cu-rich particles is the key strengthening mechanism. Therefore, it is important to investigate the precipitation behavior of Cu-rich particles in Cu-bearing RQT and DQT specimens. Figure 6 shows the TEM bright-field images of Cu-RQT and Cu-DQT, revealing the presence of precipitates of approximate radius 10 nm, and the selected area’s diffraction pattern indicates that the precipitate had Cu-rich particles. STEM was also conducted to illustrate the distribution and quantity of the Cu-rich particles, and the results are shown in Figure 7. Nanoscale precipitates can be observed in both Cu-RQT and Cu-DQT steels and are mainly distributed in lath boundaries. The number density of Cu-rich particles in the DQT steel was obviously higher than that in the RQT steel, indicating the promotion of Cu-rich particle precipitation as a result of the DQ process.

## 4. Discussion

### 4.1. The Common Effect of DQ on NiCrMo and Cu-Bearing Steels

Generally, high strength can be achieved by a combination of solid solution strengthening, grain refinement strengthening, dislocation strengthening, and precipitation strengthening. In this study, both NiCrMo-DQT and Cu-DQT specimens exhibited higher strength than NiCrMo-RQT and Cu-RQT specimens: The yield strength of NiCrMo-DQT was 106 MPa higher than that of NiCrMo-RQT, while the yield strength of Cu-DQT was 159 MPa higher than that of Cu-RQT.

**Figure 8:**
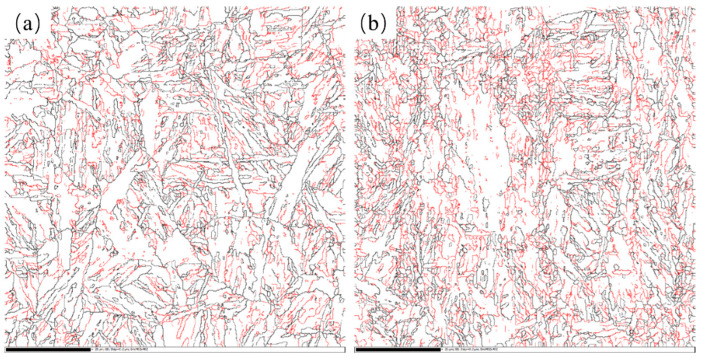
Grain boundary maps of (**a**) NiCrMo-RQT steel and (**b**) NiCrMo-DQT steel. Red boundaries: 2° < θ < 15°, black boundaries: θ > 15°.

For NiCrMo steels, since the finish rolling temperature was 780 °C, the second stage of rolling was mainly recrystallization rolling, leading to significant grain refinement in the rolled microstructure. The fine grains remained after the subsequent direct quenching. Thus, the prior austenite grain size of NiCrMo-RQ and NiCrMo-DQ steels were 14.4 μm and 8.8 μm, respectively. The tempered microstructure was further characterized by EBSD to study the effective grain size. The grain boundary maps are presented in Figure 8. The effective grain size of NiCrMo-RQT and NiCrMo-DQT steels were determined to be 3.1 μm and 2.4 μm. Meanwhile, a higher dislocation density was observed in NiCrMo-DQ and NiCrMo-DQT steels, and the dislocation was generated during non-recrystallization rolling in the second stage of the rolling process. NiCrMo-DQ steels inherited the dislocation after direct quenching and exhibited a higher level of dislocation density than NiCrMo-RQ steels, which underwent recrystallization during the austenitizing process. Though there was a certain extent of dislocation recovery during the tempering process, NiCrMo-DQT steels still exhibited a higher dislocation density than NiCrMo-RQT steels due to the previous treatment (Figure 5).

The yield strength is predicted using the following equation:

(4) $$\sigma_{y}=\sigma_{0}+\sigma_{S}+\sigma_{G}+\sigma_{D}+\sigma_{P}$$

where *σ_y_* is the yield strength in MPa, *σ*_0_ is the Peierls stress, *σ_S_* is the solid solution strengthening, *σ_G_* is the grain boundary strengthening, *σ_D_* is the dislocation strengthening, and σ_P_ is the precipitation strengthening. Since the chemical composition of NiCrMo-DQT and NiCrMo-RQT steels and their precipitation behavior are nearly the same, the σ_S_ and σ_P_ values of the two steels are considered the same. The contribution of grain refinement strengthening and dislocation strengthening can be quantitatively analyzed using the following equations:

(5) $$\sigma_{G}=K_{G}d_{e}^{1/2}$$

(6) $$\sigma_{D}=\alpha Gb\rho_{d}^{1/2}$$

where *K_G_* is a constant taken as 14.8 MPa mm^1/2^ [22], *d_e_* is the effective grain size, α is a constant equal to 0.38 [23], *G* is the shear modulus of α-Fe (80.65 GPa), *b* is the Burgers vector (0.2485 nm), and *ρ_d_* is the dislocation density. The *σ_G_* and *σ_D_* values of NiCrMo-DQT were calculated to be 36 MPa and 67 MPa higher than those of NiCrMo-RQT, leading to a total yield strength difference of 103 MPa. The calculated yield strength difference shows excellent consistency with the tested value (106 MPa), confirming that the increase in strength resulting from DQ is mainly attributed to the grain refinement strengthening and dislocation strengthening.

**Figure 9:**
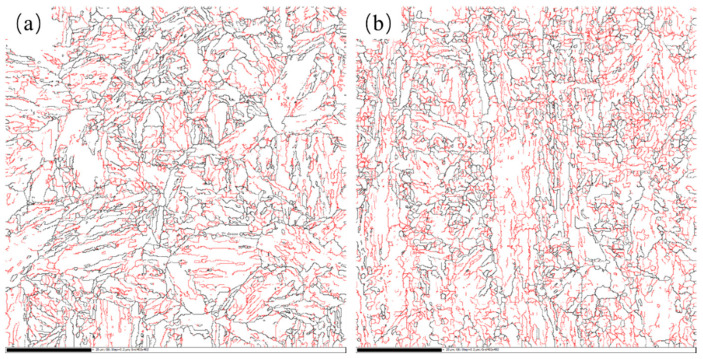
Grain boundary maps of (**a**) Cu-RQT steel and (**b**) Cu-DQT steel. Red boundaries: 2° < θ < 15°, black boundaries: θ > 15°.

The grain refinement strengthening and dislocation strengthening were also found in the Cu-bearing steels after the DQ process. The grain boundary maps are presented in Figure 9. The effective grain sizes of Cu-RQT and Cu-DQT steels were determined to be 2.7 μm and 2.3 μm. According to Equations (5) and (6), the differences in *σ_G_* and *σ_D_* between the Cu-RQT and Cu-DQT steels were calculated to be 25 MPa and 45 MPa, and the total difference value was 70 MPa. Compared with the tested yield strength difference value (159 MPa), there is a difference of 89 MPa, which is attributed to precipitation strengthening in the Cu-bearing steel.

### 4.2. The Different Effect of DQ on NiCrMo and Cu-Bearing Steels

As discussed above, only the Cu-bearing steel’s strength was improved by precipitation strengthening through the DQ process. An investigation of the substructure evolution provides some evidence for the contribution of carbides and Cu-rich particles to strength enhancement. A previous study revealed that Cu-rich particles nucleate preferentially at lath boundaries, followed by at dislocations and in the matrix [24]. The refined grain (Figure 2) and high-density dislocation (Figure 3) of Cu-DQ provide more nucleation sites for Cu-rich particles and make Cu diffusion easier during the tempering process. As a result, Cu-rich particles’ precipitation is promoted, and more Cu-rich particles are observed in the Cu-DQT steel (Figure 6 and Figure 7). Previous studies reported that Cu-rich particles can promote alloy carbides’ precipitation by acting as nucleation sites [25,26,27]. In this study, (Fe, Mn, Cr, Mo, Ni)3C carbides’ mass fraction increased with the increasing Cu-rich particles’ precipitation, revealing the synergistic enhancement of strength by carbides and Cu-rich particles in the Cu-DQT steels.

Kan [28] reported that DQT steels exhibited higher low-temperature toughness than RQT steels. The toughness was successfully increased by the refined grain size and the transition from cementite to alloy carbides. For the experimental steels, the NiCrMo-DQT steels manifested low-temperature toughness, similar to NiCrMo-RQT, while Cu-DQT steels manifested lower low-temperature toughness than Cu-RQT steels. Since the transition from cementite to alloy carbides was hardly found in the present study, this toughness difference may demonstrate the competitive relationship between the toughness loss resulting from the increasing dislocation density and the toughness enhancement resulting from grain refinement in DQ steels. The rolling parameters, including the reduction ratio and the finish rolling temperature, play a critical role in the low-temperature toughness of DQ steels.

## 5. Conclusions

The effects of direct quenching on two types of 590 MPa grade steel, composed of NiCrMo steel and Cu-bearing steel, were studied. The results are summarized as follows:

(1) Through controlled rolling and direct quenching, grain refinement and an increase in the dislocation density were observed in both NiCrMo-DQ and Cu-DQ steels. Equiaxed grains were observed in RQ steels, while deformed and elongated grains were observed in DQ steels.
(2) After appropriate tempering, NiCroMo steels exhibited a tempered martensite microstructure, whereas Cu-bearing steels exhibited a bainite microstructure. For Cu-DQT steels, the precipitation behavior of Cu particles and alloy carbides was promoted by grain refinement and the high-density dislocation during tempering.
(3) NiCrMo-DQT steels achieved high strength through grain refinement strengthening and dislocation strengthening and exhibited a yield strength of 809 MPa (106 MPa higher than NiCrMo-RQT steels), with an impact energy of 237 J at −84 °C. Cu-DQT steels achieved high strength through grain refinement strengthening, dislocation strengthening, and precipitation strengthening and exhibited a yield strength of 805 MPa (159 MPa higher than Cu-RQT steels), with an impact energy of 248 J at −84 °C.

## Figures and Tables

**Table 1:** Chemical composition of the experimental steels (wt.%).

	C	Si	Mn	Ni	Cr	Mo	V	Cu	Nb	Ti
NiCrMo	0.08	0.25	0.50	2.70	1.00	0.20	0.07			
Cu-bearing	0.04	0.25	0.60	2.40	0.60	0.35		1.50	0.03	0.02

**Table 2:** Mechanical properties of experimental steels.

	Yield Strength (MPa)	Tensile Strength (MPa)	Elongation (%)	−84 °C CVN (J)
NiCrMo-RQ	801	1078	19	196
NiCrMo-DQ	820	1107	16	199
NiCrMo-RQT	703	753	23	239
NiCrMo-DQT	809	838	20	237
Cu-RQ	743	976	16	221
Cu-DQ	759	1007	16	193
Cu-RQT	646	708	25	262
Cu-DQT	805	839	20	248

**Table 3:** Quantitative analysis of carbides in tempered steels (wt.%).

	M3C							MC					
	Fe	Mn	Cr	Mo	Ni	C*	Σ	Mo	Nb	Ti	V	C*	Σ
NiCrMo-RQT	0.577	0.028	0.161	0.010	0.0056	0.057	0.838	0.010	/	/	0.042	0.011	0.063
NiCrMo-DQT	0.577	0.028	0.158	0.011	0.0059	0.056	0.836	0.011	/	/	0.043	0.011	0.066
Cu-RQT	0.302	0.016	0.052	0.011	0.0042	0.028	0.413	0.011	0.025	0.016	/	0.0086	0.061
Cu-DQT	0.333	0.019	0.056	0.012	0.0033	0.030	0.454	0.012	0.027	0.016	/	0.0090	0.064

## Data Availability

Data are contained within the article.
